# Cardiovascular health through a sex and gender lens in six South Asian countries: Findings from the WHO STEPS surveillance

**DOI:** 10.7189/jogh.12.04020

**Published:** 2022-02-26

**Authors:** Rubee Dev, Valeria Raparelli, Louise Pilote, Zahra Azizi, Karolina Kublickiene, Alexandra Kautzky-Willer, Maria Trinidad Herrero, Colleen M Norris, the GOING-FWD Consortium

**Affiliations:** 1 Faculty of Nursing, University of Alberta, Edmonton, Alberta, Canada; 2 Department of Translational Medicine, University of Ferrara, Ferrara, Italy; 3 University Center for Studies on Gender Medicine, University of Ferrara, Ferrara, Italy; 4 Research Institute of McGill University Health Centre, Division of Clinical Epidemiology McGill University, Montreal, Quebec, Canada; 5 Centre for Outcomes Research and Evaluation, McGill University Health Centre Research Institute, Montreal, QC, Canada; 6 Department of Clinical Science, Intervention & Technology (CLINTEC), Section for Renal Medicine, Karolinska Institute and Karolinska University hospital, Stockholm, Sweden; 7 Department of Internal Medicine III, Division of Endocrinology and Metabolism, Gender Medicine Unit, Medical University of Vienna, Vienna, and Gender Institute, Iapura, Gars am Kamp, Austria; 8 Clinical & Experimental Neuroscience (NiCE-IMIB-IUIE), School of Medicine. University of Murcia, Murcia, Spain

## Abstract

**Background:**

Sex and gender-based differences in cardiovascular health (CVH) has been explored in the context of high-income countries. However, these relationships have not been examined in low- and middle-income countries. The main aim of this study was to examine how sex and gender-related factors are associated with cardiovascular risk factors of people in South Asian countries.

**Methods:**

We conducted a retrospective analysis of the World Health Organization’s “STEPwise approach to surveillance of risk factors for non-communicable disease” or “STEPS” from six South Asian countries, surveys conducted between 2014-2019. The main outcomes were CVH as measured by a composite measure of STEPS-HEART health index (smoking, physical activity, fruit and vegetable consumption, overweight/obesity, diabetes and hypertension), values ranging from 0 (worst) to 6 (best or ideal) and self-reported occurrence of cardiovascular disease (ie, heart attack and stroke). Multivariate linear and logistic regression models were performed. Multiple imputation with chained equations was performed.

**Results:**

The final analytic sample consisted of 33 106 participants (57.5% females). The mean STEPS-HEART index score in the South Asian population was 3.43 [SD: 0.92]. Female sex (β: 0.05, 95% confidence interval (CI) = 0.01-0.08, *P* < 0.05) was significantly associated with better CVH compared to males. Being married (β_male_ = -0.30, 95% CI = -0.37, -0.23 vs β_female_ = -0.23, 95% CI = -0.29, -0.17; *P* < 0.001) and having a household size ≥5 (β_male_ = -0.15, 95% CI = -0.24, -0.06 vs β_female_ = -0.11, 95% CI = -0.16, -0.04; *P* < 0.01) were associated with poorer CVH, more so in males. Being married was also associated with high risk of CVD (OR_male_ = 2.54, 95% CI = 1.68-3.86, *P* < 0.001 vs OR_female_ = 1.19, 95% CI = 0.84-1.68, *P* = 0.31), significant in males.

**Conclusions:**

Among the South Asian population, being female may be advantageous in having an ideal CVH. However, gender-related factors such as marital status and large household size were associated with poorer CVH and greater risk of CVD, regardless of sex.

As a result of an ongoing epidemiological transition, there is a rise in the burden of cardiovascular disease (CVD) in low-and middle-income countries (LMICs), accounting for nearly 80% of global CVD deaths [1]. Countries in South Asia classified as LMICs (ie, India, Pakistan, Bangladesh, Nepal, Sri Lanka, Afghanistan, Maldives, and Bhutan), represent more than a quarter of (1.8 billion-23%) the world population with about 60% of the global burden of CVD [2]. The burden of CVD in South Asia is increasing rapidly, accounting for 27% of deaths [3]. The prevalence of hypertension, one of the strong risk-factors for CVD is highest among women from South Asia [4]. One-third of the population in South Asia develops CVD during their lifetime [5], making it a major public health and clinical problem. Although considerable diversity exists between countries in South Asia, all South Asians have markedly higher risk of CVD than other ethnicities [3,6]. The rise in CVD might reflect the intersectionality of poor health systems, behavioral factors, genetic factors, and gender factors in South Asia.

In fact, while the burden of CVD and trends in South Asia are mainly reported by sex and age disaggregated data, the impact of gender, defined as the socially constructed roles and norms of women and men [7] has been largely neglected. Beyond traditional CV risk factors (ie, hypertension diabetes, dyslipidemia and smoking) highly and alarmingly prevalent in South Asian countries [8-10]; the effect of non-traditional risk factors such as gender roles or behaviors are understudied. Existing literature on South Asian countries report mainly on women’s empowerment and related gender constructs, such as ‘women’s autonomy’ and ‘women’s decision-making power’ as the major and most frequently used gender-related factors [11]. Furthermore, these factors have been mainly used to examine their impact on the use of family planning methods or maternal and child health outcomes [12]. Additional research on identification and examination of gender-related factors that could impact the cardiovascular health (CVH) of individuals in South Asia is needed. Hence, our main objective was to examine how sex and gender-related factors are associated with the cardiovascular risk factors of South Asians.

## METHODS

### Study design

This was a cross-sectional study using a population-based household survey of adults aged 15-69 years. The data came from the World Health Organization’s (WHO) “STEPwise approach to surveillance of risk factors for non-communicable disease” or “STEPS” survey [13]. STEPS is a household survey tool, conceptualized and developed by the WHO for collecting, analyzing, and disseminating data on risk factors of non-communicable diseases and associated health system response at the population level. STEPS data from 6 South-Asian countries including Nepal, Bangladesh, Pakistan, Afghanistan, Bhutan, and Sri Lanka were merged together to represent the South-Asian population.

### Study participants and sampling

A total of 33 106 survey participants (women:19028, 57.5%) were included in the study. The surveys were conducted between 2014-2019. The inclusion criteria consisted of participants of working age 18–69 years. Systematic random sampling of households through multistage stratified cluster-sampling was used to form the cohorts and data was collected through in-person interviews, physical measurements, and biochemical measurements. The most current survey data were obtained from the WHO “NCD Microdata Repository” for each country in South Asia following the required standard procedure and obtaining data access approval.

### Outcome variables

The main outcome variables were CVH as measured by a composite measure of CVH and the self-reported occurrence of CVD (ie, heart attack and stroke). We created a composite measure of the CVH using the Cardiovascular Health in Ambulatory Care Research Team (CANHEART) health index [14], a measure of ideal CVH composed of 6 cardiometabolic health metrics also referred to as behavioral risk-factors (ie, smoking, physical activity, fruit and vegetable consumption, overweight/obesity, diabetes and hypertension). The presence of each ideal health behavior or factor was assigned a value of 1 for an ideal state and 0 for a nonideal state. The number of ideal metrics were then summed to create the index for the South Asian population. The index ranges from 0 (worst) to 6 (best or ideal), with higher scores reflecting ideal CVH. Respondents were considered to be in an ideal state, if they were: not a current smoker, their moderate physical activity in leisure time exceeded >10 minutes, their daily fruit and vegetable consumption was ≥5 servings per day, were not overweight or obese ie, (body mass index (BMI)<25), and self-reported no hypertension or diabetes diagnosed by health professional **(**Table S1 in the **Online Supplementary Document****)**.

### Explanatory variables

Age of the respondents was recorded as a continuous variable in the data set and was categorized into six groups: <20, 20-29, 30-39, 40-49, 50-59, and 60-69 years. Self-reported biological-sex and gender-related variables (education level, marital status, work status, and household size) available in the data set were identified and chosen based on the recently published methodology by GOING-FWD investigators who identified how to measure gender in retrospective cohort studies [15]. Biological sex in this study was defined as male or female; education level was categorized into three groups: less than secondary, secondary, and post-secondary; marital status was categorized into three groups: single/never married, widow/separated/divorced, and married/common law; work status was categorized into three groups: employed/self-employed/retired, unemployed/student/non-paid, and homemaker; and household size was categorized into five groups: 1, 2, 3, 4, and ≥5 people in the household. All categorizations were based on the STEPS manual [16].

### Statistical analysis

Frequency tabulations were conducted to describe the data followed by the contingency table analyses to examine the identified gender-related factors associated with biological sex. Gender-related factors were reported as frequency and percentages. A multivariable linear regression model was used to examine the association between sex, gender-related factors, and a composite measure of CVH as a continuous dependent variable. A multivariable logistic regression model was used to examine the association between sex, gender-related factors, and CVD as a binary outcome (ie, history of heart attack and/or stroke) among the South Asian population. All tests were two-sided and the α threshold was set at 0.05. Analyses were conducted using Stata version 15.0 (Stata Corporation, College Station, TX, USA).

### Multiple imputation for missing data

To reduce potential bias created by missing data, multiple imputation methods were used, through the *mi impute* command on Stata. Multiple Imputation using Chained Equations (MICE) method [17,18] in which missing data are imputed based on the distribution of other variables were used to impute data for all variables (education level: 8.9%, marital status: 15.7%, household size: 24.9%) with missing information, and also included complete variables (age, sex, work status) to generate 10 imputed data sets for use in the analyses.

## RESULTS

### Gender-related factors

Nearly half of the study population (47%) was from Bangladesh and Pakistan. The proportion of male and female individuals was similar across all the South-Asian countries. The mean age of the population was 39.3 years (standard deviation [SD]: 13.5), the majority (69.8%) in the age group 20-49 years. Amongst gender-related variables, females had more likely less than secondary education than males (67.2% vs 60.2%). The majority of the respondents reported being married (83.7%) and more than half (53.6%) reported having household size of two or three people living in the household. The proportion of females who reported being a homemaker was vastly greater than for males (69.7% vs 5.2%) (**Table 1**).

**Table 1:** Descriptive characteristics of survey respondents, by biological sex (n = 33 106)

	N*	Overall	Biological sex	
**Male (N = 14 078)**	**Female (N = 19 028)**
**n (%) or mean (SD)**	**n (%) or mean (SD)**	**n (%) or mean (SD)**
**Demographic variables**				
**Country/Survey year**	33106			
Nepal, 2019		5593 (16.9)	1998 (14.2)	3595 (18.9)
Bangladesh, 2018		8185 (24.7)	3804 (27.0)	4381 (23.0)
Afghanistan, 2018		3952 (11.9)	2022 (14.4)	1930 (10.1)
Sri Lanka, 2014-2015		5188 (15.7)	2030 (14.5)	3158 (16.6)
Pakistan, 2013-2014		7366 (22.3)	3150 (22.3)	4216 (22.2)
Bhutan, 2014		2822 (8.5)	1074 (7.6)	1748 (9.2)
**Age (in years)**	33079	39.3 [13.5]	40.9 [13.9]	38.1 [13.1]
**Age categories**	33076			
<20		1670 (5.1)	683 (4.9)	987 (5.2)
20-29		7341 (22.2)	2732 (19.4)	4609 (24.2)
30-39		8830 (26.7)	3417 (24.3)	5413 (28.5)
40-49		6917 (20.9)	3057 (21.7)	3860 (20.3)
50-59		4880 (14.8)	2345 (16.7)	2535 (13.3)
60-69		3438 (10.4)	1828 (13.0)	1610 (8.5)
**Gender variables:**				
**Education level**	30198			
Less than secondary		19357 (64.1)	7956 (60.2)	11401 (67.2)
Secondary		8756 (29.0)	4081 (30.9)	4675 (27.6)
Post-secondary		2085 (6.9)	1190 (9.0)	895 (5.3)
**Marital status**	27910			
Single/Never married		3083 (11.1)	1730 (14.4)	1353 (8.5)
Widow/Separate/Divorce		1456 (5.2)	170 (1.4)	1286 (8.1)
Married/Common in law		23371 (83.7)	10142 (84.2)	13229 (83.4)
**Work status**	32999			
Employed		15131 (45.9)	11357 (81.1)	3774 (19.9)
Unemployed		3904 (11.8)	1914 (13.7)	1990 (10.5)
Homemaker		13964 (42.3)	732 (5.2)	13232 (69.7)
**Household size**	24861			
1		1713 (6.9)	521 (5.1)	1192 (8.2)
2		7914 (31.8)	3308 (32.3)	4606 (31.5)
3		5418 (21.8)	1869 (18.2)	3549 (24.3)
4		3997 (16.1)	1964 (19.2)	2033 (13.9)
≥5		5819 (23.4)	2591 (25.3)	3228 (22.1)
**Behavioral risk-factors**				
**Smoking**	33096			
Smoker		5093 (15.4)	4351 (30.9)	742 (3.9)
Non-smoker		28003 (84.6)	9725 (69.1)	18278 (96.1)
**Overweight/obesity**	31418			
BMI<25		11996 (38.2)	4638 (33.9)	7358 (41.5)
BMI≥25		19422 (61.8)	9055 (66.1)	10367 (58.5)
**Leisure physical activity**	33076			
>10 min activity		30079 (90.9)	12081 (85.9)	17998 (94.7)
≤10 min activity		2997 (9.1)	1980 (14.1)	1017 (5.4)
**Fruit and vegetable consumption**	24323			
≥5 servings/d		2928 (12.0)	1247 (11.7)	1681 (12.3)
<5 servings/d		21395 (87.9)	9425 (88.3)	11970 (87.7)
**Hypertension**	33073			
Yes		5215 (15.8)	1794 (12.8)	3421 (17.9)
No		27858 (84.2)	12266 (87.2)	15592 (82.0)
**Diabetes**	33072			
Yes		1815 (5.5)	781 (5.6)	1034 (5.4)
No		31258 (94.5)	13279 (94.5)	17979 (94.6)
**Outcome variables**				
**Heart attack or stroke**	33071			
Yes		2134 (6.5)	943 (6.7)	1191 (6.3)
No		30937 (93.5)	13116 (93.3)	17821 (93.7)
**STEPS-HEART Index†**	22993			
0		37 (0.2)	29 (0.3)	8 (0.1)
1		549 (2.4)	248 (2.4)	301 (2.4)
2		2669 (11.6)	1282 (12.4)	1387 (10.9)
3		8132 (35.4)	3814 (36.9)	4318 (34.2)
4		9519 (41.4)	3934 (38.0)	5585 (44.2)
5		1957 (8.5)	967 (9.3)	990 (7.8)
6		130 (0.6)	75 (0.7)	55 (0.4)

BMI – body mass index, SD – standard deviation

*Number of observations with complete information

†Cardiovascular health index for South Asian population

### Cardiovascular health

The mean CVH index score was 3.43 (standard deviation (SD) = 0.92] with median of 4 (interquartile range (IQR) = 3-4). The score was significantly higher in females compared to males (3.45 vs 3.41, *P* < 0.001). Majority of the respondents reported index score of 3 and 4, with females reporting greater proportion of better CVH (78.4% vs 74.9%). Amongst ideal behavioral factors, as compared with males, females had higher ideal scores: smoking (96.1% vs 69.1%), overweight/obesity (41.5% vs 33.9%), physical activity (94.7% vs 85.9%), and fruit and vegetable consumption (12.3% vs 11.7%). The self-reported prevalence of hypertension was greater among females than males (17.9% vs 12.8%), while the prevalence of diabetes (5.4% vs 5.6%) was similar across both sexes **(****Table 1****)**. Respondents who were homemakers (odds ratio (OR) = 40.73, 95% confidence interval (CI) = 36.39-45.59) or married (OR = 1.39, 95% CI = 1.21-1.60) were more likely to be females. While respondents with higher education level were less likely to be females (OR = 0.61, 95% CI = 0.52-0.71) (*P* < 0.001 for all) **(****Table 2****)**.

**Table 2:** Multivariable logistic model for assessing association of gender-related variables with biological sex as dependent variable

Gender variables	OR (95% CI)	*P*-value
**Age**		
<20 (ref)	-	-
30-39	0.78 (0.66-0.93)	<0.01
20-29	0.61 (0.50-0.74)	<0.001
40-49	0.43 (0.35-0.53)	<0.001
50-59	0.27 (0.22-0.34)	<0.001
60-69	0.11 (0.09-0.15)	<0.001
**Education level:**		
Less than secondary (ref)	-	-
Secondary	0.58 (0.52-0.65)	<0.001
Post-secondary	0.61 (0.52-0.71)	<0.001
**Marital status:**		
Single/Never married (ref)	-	-
Widow/Separated/Divorced	11.12 (8.47-14.61)	<0.001
Married/Common in law	1.39 (1.21-1.60)	<0.001
**Work status:**		
Employed (ref)	-	-
Unemployed	2.59 (2.31-2.91)	<0.001
Homemaker	40.73 (36.39-45.59)	<0.001
**Household size:**		
1 (ref)	-	-
2	0.47 (0.39-0.56)	<0.001
3	0.59 (0.48-0.71)	<0.001
4	0.29 (0.24-0.35)	<0.001
≥5	0.36 (0.30-0.44)	<0.001

OR – odds ratio, CI – confidence interval

Female sex (β = 0.05, 95% CI = 0.01-0.08, *P* < 0.05) was significantly associated with better CVH. CVH significantly declined with increasing age regardless for sex. Respondents with post-secondary education were more likely to have a poor CVH than those with less than secondary education, more so among males (β_male_ = -0.11, 95% CI = -0.16, -0.05, *P* < 0.001 vs β_female_ = -0.08, 95% CI = -0.14, -0.01, *P* < 0.01). Married males had a poorer CVH than married females (β_male_ = -0.30, 95% CI = -0.37, -0.23 vs β_female_ = -0.23, 95% CI = -0.29, -0.17; *P* < 0.001 for both) compared to their single/never married counterparts. While female homemakers compared to employed females showed poorer CVH (β_female_ = -0.08, 95% CI = -0.11, -0.04, *P* < 0.001), male homemakers showed better CVH (β_male_ = 0.12, 95% CI = 0.04-0.18, *P* < 0.01) compared to the employed males **(****Table 3****)**.

**Table 3:** Multiple imputation linear regression for examining association between cardiovascular health with biological sex and gender-related variables in South Asian population (n = 33** **106)

Cardiovascular health (STEPS-HEART index)	Overall (n = 33 106)		Male (n = 14 078)		Female (n = 19 028)	
**Unstandardized coefficient (β)**	***P*-value**	**Unstandardized coefficient (β)**	***P*-value**	**Unstandardized coefficient (β)**	***P*-value**
**Sex (Female)**	0.05 (0.01, 0.08)	<0.05	-	-	-	-
**Age:**						
<20 (ref)	-		-		-	
20-29	-0.16 (-0.22, -0.11)	<0.001	-0.18 (-0.28, -0.09)	<0.001	-0.14 (-0.22, -0.07)	<0.001
30-39	-0.37 (-0.43, -0.32)	<0.001	-0.39 (-0.49, -0.29)	<0.001	-0.36 (-0.43, -0.28)	<0.001
40-49	-0.55 (-0.61, -0.49)	<0.001	-0.53 (-0.64, -0.43)	<0.001	-0.56 (-0.64, -0.48)	<0.001
50-59	-0.63 (-0.69, -0.57)	<0.001	-0.62 (-0.72, -0.52)	<0.001	-0.64 (-0.73, -0.55)	<0.001
60-69	-0.66 (-0.73, -0.59)	<0.001	-0.63 (-0.74, -0.53)	<0.001	-0.70 (-0.80, -0.60)	<0.001
**Education level:**						
Less than secondary (ref)	-		-		-	
Secondary	0.01 (-0.01, 0.04)	0.27	-0.03 (-0.06, 0.01)	0.10	0.05 (0.01, 0.08)	<0.01
Post-secondary	-0.09 (-0.13, -0.05)	<0.001	-0.11 (-0.16, -0.05)	<0.001	-0.08 (-0.14, -0.01)	0.01
**Marital status:**						
Single/Never married (ref)	-		-		-	
Widow/Separated/Divorced	-0.31 (-0.38, -0.24)	<0.001	-0.26 (-0.41, -0.13)	<0.001	-0.27 (-0.36, -0.19)	<0.001
Married/Common in law	-0.26 (-0.30, -0.22)	<0.001	-0.30 (-0.37, -0.23)	<0.001	-0.23 (-0.29, -0.17)	<0.001
**Work status:**						
Employed (ref)	-		-		-	
Unemployed	0.07 (0.04, 0.11)	<0.001	0.09 (0.05, 0.15)	<0.001	0.01 (-0.03, 0.06)	0.62
Homemaker	-0.03 (-0.07, 0.01)	0.05	0.12 (0.04, 0.18)	<0.01	-0.08 (-0.11, -0.04)	<0.001
**Household size:**						
1 (ref)	-		-		-	
2	-0.07 (-0.13, -0.01)	<0.05	-0.07 (-0.17, 0.02)	0.13	-0.06 (-0.13, 0.004)	0.06
3	-0.13 (-0.18, -0.08)	<0.001	-0.15 (-0.24, -0.05)	<0.01	-0.11 (-0.17, -0.04)	<0.01
4	-0.11 (-0.17, -0.06)	<0.001	-0.12 (-0.22, -0.02)	<0.05	-0.10 (-0.17, -0.04)	<0.01
≥5	-0.13 (-0.17, -0.08)	<0.001	-0.15 (-0.24, -0.06)	<0.01	-0.11 (-0.16, -0.04)	<0.01

MICE – multiple imputation using chained equations

### Cardiovascular disease

The prevalence of CVD was similar among males (6.7%, n = 943) and females (6.3%, n = 1191), which is high for the given age group, especially in females **(****Table 1****)**. The risk of CVD increased with increasing age, magnitude of effect being significantly higher for females compared to males in all age group, indicating a worrisome fact that women are catching up with the growing epidemic of CVD in the South Asian countries. The risk also increased with increase in the number of household size. Females with household size of 4 were significantly more likely to experience CVD than males (OR_female_ = 1.71, 95% CI = 1.28-2.27, *P* < 0.001 vs OR_male_ = 1.53, 95% CI = 0.98-2.38, *P* = 0.06). Females and males with secondary level education had a lower prevalence of CVD as compared to those with less than secondary education (OR_female_ = 0.74, 95% CI = 0.63-0.86, *P* < 0.001 vs OR_male_ = 0.81, 95% CI = 0.68-0.94, *P* < 0.05). Furthermore, married males (OR_male_ = 2.54, 95% CI = 1.68-3.86, *P* < 0.001) and widowed/separated/divorced females (OR_female_ = 1.75, 95% CI = 1.18-2.60, *P* < 0.01) had a higher risk of CVD compared to their single or never married counterparts **(****Table 4****)**.

**Table 4:** Multiple imputation logistic regression for examining association between cardiovascular disease with biological sex and gender-related variables in South Asian population

Cardiovascular disease (Heart attack and stroke)	Overall (n = 33 106)		Male (n = 14 078)		Female (n = 19 028)	
**OR (95% CI)**	***P*-value**	**OR (95% CI)**	***P* -value**	**OR (95% CI)**	***P* -value**
**Sex (Female)**	0.96 (0.85-1.09)	0.57	-	-	-	-
**Age:**						
<20 (ref)	-		-		-	
20-29	1.20 (0.85-1.71)	0.29	0.78 (0.46-1.33)	0.37	1.61 (1.01-2.59)	<0.05
30-39	1.82 (1.27-2.59)	<0.01	1.16 (0.67-2.02)	0.57	2.45 (1.52-3.94)	<0.001
40-49	2.16 (1.51-3.09)	<0.001	1.33 (0.76-2.31)	0.31	3.01 (1.87-4.86)	<0.001
50-59	2.68 (1.87-3.83)	<0.001	1.98 (1.14-3.43)	<0.05	3.18 (1.96-5.17)	<0.001
60-69	3.37 (2.35-4.84)	<0.001	2.57 (1.48-4.45)	<0.01	3.76 (2.29-6.18)	<0.001
**Education level**						
Less than secondary (ref)	-		-		-	
Secondary	0.76 (0.68-0.85)	<0.001	0.81 (0.68-0.94)	<0.05	0.74 (0.63-0.86)	<0.001
Post-secondary	0.90 (0.74-1.10)	0.32	1.01 (0.78-1.29)	0.93	0.75 (0.54-1.05)	0.1
**Marital status:**						
Single/Never married (ref)	-		-		-	
Widow/Separated/Divorced	2.23 (1.61-3.08)	<0.001	1.54 (0.65-3.63)	0.31	1.75 (1.18-2.60)	<0.01
Married/Common in law	1.76 (1.35-2.29)	<0.001	2.54 (1.68-3.86)	<0.001	1.19 (0.84-1.68)	0.31
**Work status:**						
Employed (ref)	-		-		-	
Unemployed	1.09 (0.92-1.28)	0.31	1.26 (1.02-1.55)	<0.05	0.92 (0.71-1.21)	0.57
Homemaker	1.00 (0.88-1.14)	0.97	0.56 (0.40-0.79)	<0.01	1.08 (0.93-1.27)	0.28
**Household size:**						
1 (ref)	-		-		-	
2	1.37 (1.03-1.82)	<0.05	1.48 (0.95-2.31)	0.08	1.28 (0.94-1.76)	0.11
3	1.61 (1.25-2.08)	<0.001	1.47 (0.95-2.29)	0.08	1.65 (1.23-2.21)	<0.01
4	1.61 (1.26-2.05)	<0.001	1.53 (0.98-2.38)	0.06	1.71 (1.28-2.27)	<0.001
≥5	1.86 (1.43-2.42)	<0.001	2.33 (1.51-3.62)	<0.001	1.51 (1.12-2.04)	<0.01

OR – odds ratio, CI – confidence interval

## DISCUSSION

In this cross-sectional, population-based study in a South Asian population, we found that female sex was associated with a better CVH and a slightly lower prevalence of CVD (heart attack and stroke) than male sex. Given the CVD epidemic in South Asian countries, it is noteworthy that women are catching up with the similar prevalence as of men. Independent of sex, being married or widowed and a larger household size were associated with poorer CVH and a higher prevalence of CVD. Moreover, a higher level of education was associated with a lower prevalence of CVD but was not associated with better CVH.

This study extends the prior literature in several ways. We reported that female sex was associated with a better CVH and a lower prevalence of heart disease among individuals from LMICs similarly to what we recently observed among Canadians and Austrians (ie, high-income countries) [19]. Lower prevalence of CVD among women was also reported in a study conducted in 27 high-income, middle-income, and low-income countries [20]. Smoking remains a key risk factor for the development of CVD in both males and females. Burke et al. demonstrated that male smokers have a 2-fold increased risk of CVD while females 1.5-fold increased risk [21]. In our study, there was a lower prevalence of females who smoked than males, which may have led to a lower CVH among males. Contrary to these findings, a study conducted in rural Uganda showed female sex to be associated with poor CVH despite females having better indices for ideal CVH behaviors [22]. One possible cause for these differences may be explained by different indices and units used for creating the STEPS-HEART index. Nevertheless, these findings highlight the importance of identifying sex-specific risk factors that disproportionately contributes toward CVD risk in males and females through targeted public health programs.

Earlier reviews of the evidence have shown that being married is associated with lower risk of CVD and better CVH [23-25]. The protective effect of being married is in line with findings from another study conducted in multiple US and international cohorts that showed respondents who were unmarried including those who were divorced, separated, or never married, had increased rates of adverse cardiovascular events; and importantly that the association was stronger in males [24,26]. Marriage is traditionally seen a form of social support that may decrease the prevalence of risky health behaviors, leading to a beneficial effect on health [27]. However, an inverse association was observed in our South Asian population that demonstrated that being single or never married was associated with a lower risk of CVD compared to their married or divorced counterparts, more in males. One of the reasons behind this contrast finding could be less physical activity among married male participants in South Asian countries [28]. Further understanding of the reasons behind such conflicting finding in LMICs and high-income countries is very much needed to better tailor gender-sensitive interventions and it might be rooted on culture-specific structure of the society and family in South Asia.

The impact of household size on the risk of CVD is sparse in the literature. In the context of South Asia, household size could be a marker of socioeconomic status, which has been clearly linked with cardiovascular outcomes [29]. Regardless of the household size, the majority of the families in LMICs are single-earner families making it unaffordable for them to adopt healthy lifestyle behaviors related with preventing CVDs such as consumption of two serving of fruits and three servings of vegetables per day, as evident in only 12% of the respondents in our study. Similar to the findings of our study, a study conducted in 28 LMICs reported only 18% of the individuals met the WHO recommendation of fruits and vegetables consumption [30]. Observed inequities in such lifestyle behavior in South Asian countries could have led to the increased risk of CVD among the respondents with increasing household size. Policies to reduce behavioral inequities must include strategies to overcome barriers to availability and affordability, especially for those with lower levels of household income.

Education level of respondents was found to be one another important predictor of CVH and CVD among South Asian population. Respondents with high level of education were at lower risk of CVD compared to those with low level of education. This is in line with study findings that reported higher hazards of major cardiovascular events among those with low level of education compared to high level of education in all levels of income countries studied, but much more pronounced in LICs (HR = 2.23) [29]. Level of education maybe a proxy for health literacy that has been linked to empowerment, use of health services, ability to find and understand information correctly, and ability to participate in a discussion with health care providers [31]. Of note in our study respondents with a higher level of formal education did not demonstrate better CVH. Modifiable risk factors such as BMI and physical activity are shown to mediate the association between education and CVH [32]. In contrast to the inverse association between BMI and education level in HICs [33], LMICs show direct association between higher BMI among individuals with high level of education [34], impacting their overall CVH index. Future studies could explore the association between BMI and education level specific to the context of South Asian countries where the burden of CVD is rising due to an epidemiologic transition.

This study has several strengths. The data were collected from a nationally representative survey of the South Asian population that included data on a range of lifestyle risk factors making it possible to create a STEPS-HEART health index. With this we were able to expand our sex stratified analyses to examine a range of gender-related factors associated with the CVH. Moreover, our study included the most recent available data for each country. However, there are also some limitations that need to be considered when interpreting the findings of this study. First, we were restricted to the number of gender-related factors that were measured in the survey. Second, even though some gender-related factors such as annual household income and home stress were available, we were not able to use it due to: (i) missing data in the data sets for three countries (Nepal, Bangladesh, and Afghanistan) and (ii) harmonization issues due to significantly different measures used in different countries. Third, while we included data on six South Asian countries, nearly half of the respondents were from Bangladesh and Pakistan. Fourth, there could be a potential measurement error in our study outcome due to the self-reported nature of the survey. Given that health literacy and health systems are often weak in many South Asian countries [4], it is possible that CVD might have been underreported. Hence, the findings should be interpreted with caution. Fifth, the composite measure of CVH did not include abnormal lipid level (a key CVD risk factor) due to the high proportion (>50%) of missing observations on a variable that would have biased the results [35,36]. Finally, we were not able to include data from India, which is the largest country in South Asia in our analyses, as no national data set is available for the country. However, it is interesting to note that India only collects region-specific (sub-national) data due to the recognized wide variation across this geographic region [37].

## CONCLUSIONS

In conclusion, our study demonstrated that being female may be advantageous in terms of having ideal heart factors leading to better CVH and a lower risk of CVD in the context of South Asian population but gender-related factors such as marital status and large household size disproportionately impact the health of both sexes. The findings of this study highlight the importance of sex-specific preventive programs to target both males and females who are disproportionality impacted by the gender-related factors. Furthermore, the contrasts identified between sex, gender-related factors and CVD risk and outcomes suggest that while age/sex-stratified analyses are an imperative first step, health outcomes research going forward must be rooted in an intersectional understanding of the influence of age, sex, gender, culture, ethnicity and country, on CVD risk factors and CV health [38].

## Additional material


Online Supplementary Document
