# Supplementary material for: Cardiovascular health through a sex and gender lens in six South Asian countries: Findings from the WHO STEPS surveillance
Source: J Glob Health. 2022 Feb 26;12:04020. doi: 10.7189/jogh.12.04020 (PMC8876159; doi:10.7189/jogh.12.04020)
Supplement: Online Supplementary Document [file jogh-12-04020-s001.pdf]

Appendix

Table 1. Definition of each indicators and outcomes

**Smoking**
Currently smoking
**Overweight/obesity**
BMI > 25 kg/m^2 (Calculated using measured height and weight)
**Hypertension**
Self-reported hypertension diagnosed by a health care professional
**Diabetes**
Self-reported diabetes diagnosed by a health care professional
**Fruit and vegetable consumption**
Fruit and vegetables consumed ≥ 5 times per day
**Physical activity**
Moderate physical activity (days per week)
**Heart attack and stroke**
Self-reported heart attack and stroke diagnosed by a health care professional
